# Clinico-Epidemiologic Characteristics of Patients Reported in the Mycotic Infections in COVID-19 Registry

**DOI:** 10.4269/ajtmh.22-0503

**Published:** 2022-12-19

**Authors:** Rohan Maini, Neha Saini, Kranti Bhavana, Bhartendu Bharti, Shweta Walia, Neetu Kori, Sushila Kataria, Pooja Sharma, Vikas Deswal, Kavya Atluri, Yatin Sethi, Charuta Mandke, Mayank Chansoria, Sumit Rawat, Rajani Bhat, Ameet Dravid, Chandan Baranwal, Nirmal Sarkar, Sunit Jariwala, Yoram Puius, Shitij Arora, for the MUNCO Registry

**Affiliations:** ^1^ Internal Medicine, Montefiore Medical Center, Albert Einstein College of Medicine, Bronx, New York;; ^2^ ENT, All India Institute of Medical Sciences, Patna, India;; ^3^ Opthamology, Maharaja Yeshwantrao Hospital, Indore, India;; ^4^ Medanta Institute of Medicine and Research, Delhi, India;; ^5^ Department of Bioinformatics, University of California, Los Angeles, California;; ^6^ ENT, Venkateshwara Hospital, Dwarka, New Delhi, Delhi;; ^7^ Opthalmology, HinduHrudaySamrat Balasaheb Thackarey Medical College, Mumbai, India;; ^8^ Emergency Medicine, Netaji Subhash Chandra Bose Medical College, Jabalpur, India;; ^9^ Microbiology, Bundelkhand Medical College, Sagar, India;; ^10^ Consultant Pulmonologist, Bangalore, India;; ^11^ Infectious Diseases, Noble Hospital, Pune, India;; ^12^ ENT, Nepal Mediciti Hospital, Karyabinayak, Nepal;; ^13^ Pulmonology and Chest Medicine, Sarkari Kormochari Hospital, Dhaka, Bangladesh

## Abstract

We update results from the Mycotic Infections in COVID-19 (MUNCO) Registry, May–September 2021. Data collection from May to September 2021 yielded 728 cases from India, Nepal, Bangladesh, Thailand, and the United States. The cases consisted of mostly mucormycosis (97.6%), primarily rhinocerebral, and were analyzed to investigate clinical characteristics associated with negative outcomes. Patients were mostly diabetic (85%) and male (76%), with significant mortality (11.7%). All patients received treatment of coronavirus disease 2019 (COVID-19) as well as antifungal treatment. The crude mortality rate was 11.3% for mucormycosis and 22.7% formixed infections. This study demonstrates the utility of online databases in the collection of high-caliber data.

## INTRODUCTION

The coronavirus disease 2019 (COVID-19) pandemic caused by severe acute respiratory syndrome coronavirus 2 has been associated with secondary fungal infections, notably mucormycosis, aspergillosis, and candidiasis.^1^ To identify clinical characteristics associated with poor outcomes of COVID-19–associated mycoses, we previously established the Mycotic Infections in COVID-19 (MUNCO) Registry,^2^ which accumulated 65 cases of Coronavirus-associated mucormycosis in India and South Asia in close to real time. We present here updated data describing 728 cases, now including mucormycosis, aspergillosis, and candidiasis.

## METHODS

As previously described,^2^ cases were solicited through social media and contacts at participating hospitals and entered into a deidentified, secure electronic database at https://www.covidmucor.com using REDCap.^3^ Mycoses were based on the judgment of the clinician entering the data and included all patients with histopathologically confirmed infection. The primary outcome was in-hospital mortality. Secondary outcomes were measured at the time of COVID-19 diagnosis and included hospital admission, length of admission, intensive care unit (ICU) admission, oxygen requirement, incomplete recovery, and vision loss. Incomplete recovery was defined as continued treatment at day 42, interrupted treatment, palatal perforation, stroke, or paralysis.

## RESULTS

The 728 cases were collected from May to September 2021 during predominance of the B.1.617.2 lineage (Delta variant). Cases were reported from India, Bangladesh, Thailand, and Nepal, and one came from the United States (Supplemental Figure 1).

Table 1 shows demographics and clinical characteristics of the cases, mostly CAM. Median age and body mass index were similar across all fungal isolates. Patients were predominantly male and unvaccinated, with either uncontrolled or newly diagnosed diabetes. The major fungal infection was mucormycosis (97.6%), and the sites most often involved with any fungal infection were sinuses (95.6%), eyes (58.1%), and brain (18.1%). Inflammatory marker levels were similar across all isolates.

**Table 1:** Demographics and clinical characteristics of patients with COVID-19–associated mycoses

Patient Characteristics	Mucormycosis (*N* = 711)*	*Aspergillus* (*N* = 27)*	Candida (*N* = 14)*	Mixed (*N* = 22)*†
Patient demographics				
Age, years (*N* = 728)				
Median	51	56	57	55
2–25	10 (1.4)	0 (0)	0 (0)	0 (0)
26–50	322 (45)	10 (37)	6 (42)	9 (40)
51–75	371 (52)	15 (55)	5 (35)	11 (50)
76–87	8 (1)	2 (7.4)	3 (21)	2 (9)
Sex				
Male	545 (76)	19 (70)	9 (64)	17 (77)
Female	165 (23)	8 (29)	5 (35)	5 (22)
BMI, kg/m^2^ (*N* = 721)				
Average	24.0	23.8	23.9	24.4
14–25	464 (65)	14 (51)	9 (64)	13 (59)
25–30	183 (25)	8 (29)	5 (35)	8 (36)
30–40	36 (5)	0 (0)	0 (0)	1 (4.5)
> 40	2 (0.2)	0 (0)	0 (0)	0 (0)
Comorbidities (*N* = 676)				
Diabetes	607 (85)	27 (100)	12 (85)	20 (90)
Controlled	158 (22)	5 (18.5)	0 (0)	3 (13)
Uncontrolled	342 (48)	19 (70.3)	11 (78)	16 (72)
New diagnosis	102 (14)	2 (7.4)	0 (0)	1 (9)
DKA	20 (2.8)	1 (3.7)	0 (0)	1 (9)
Prior long-term steroid	70 (9)	2 (7.4)	0 (0)	2 (9)
Asthma/COPD	16 (22)	0 (0)	1 (7)	0 (0)
IVDU	3 (0.4)	3 (11)	0 (0)	2 (9)
HIV	3 (0.4)	0 (0)	0 (0)	0 (0)
Transplant	8 (1)	0 (0)	0 (0)	0 (0)
Cancer	3 (0.4)	0 (0)	0 (0)	0 (0)
Other	171 (24)	9 (33)	10 (71)	12 (54)
Vaccination status (*N* = 711)				
Vaccinated	109 (15)	5 (18)	3 (21)	6 (27)
COVISHIELD	93 (13)	4 (14.8)	2 (14)	5 (22)
COVAXIN	13 (1.8)	0 (0)	1 (7)	1 (9)
Single dose	83 (11)	1 (3.7)	2 (14)	4 (18)
Double dose	25 (3)	3 (11)	1 (7)	2 (9)
Unvaccinated	602 (84)	22 (81)	11 (78)	16 (72)
Site of involvement (*N* = 709)‡				
Sinus	667 (93.8)	10 (37)	1 (7.1)	–
Ophthalmic	408 (57.4)	4 (14.8)	1 (7.1)	–
Cerebral	128 (18)	0 (0)	0 (0)	–
Pulmonary	17 (2.4)	1 (3.7)	3 (21.4)	–
Gastrointestinal	10 (1.4)	1 (3.7)	0 (0)	–
Cutaneous	8 (1.1)	0 (0)	0 (0)	0
Mean C-reactive protein (normal < 3 mg/L)	74.8	45.1	67.6	71.5
Mean ferritin (normal 12–1,300 ng/mL)	605	575.6	753	1,060

– = not available; BMI = body mass index; COPD = chronic obstructive pulmonary disease; DKA = diabetic ketoacidosis.

* Values are expressed as n (%) unless otherwise indicated.

† Totals may add up to more than 100% because of coinfection by multiple fungi.

‡ Totals may add up to more than 100% because of infection at multiple sites.

The average time between diagnosis of COVID-19 and fungal infection was 24.5 days. A total of 438 patients (60.8%) were hospitalized for COVID-19 or related indications for a mean of 12.8 days, and 107 patients (24.9%) were admitted to the ICU (Table 2). The *Aspergillus* group had the longest length of stay. Although most patients did not require oxygen, 87 (12.6%) required a high-flow nasal cannula, 68 (9.9%) required a non-rebreather, and 25 (3.6%) required a ventilator.

**Table 2:** Treatments and outcomes of patients with COVID-19–associated mycoses

Treatment data	Mucor (*N* = 711)*	*Aspergillus* (*N* = 27)*	Candida (*N* = 14)*	Mixed (*N* = 22)*†
COVID-19 treatment				
Favipiravir	163 (22.9)	10 (37)	6 (42.9)	11 (50)
Remdesivir	266 (37.4)	17 (63)	12 (85.7)	15 (68.2)
Corticosteroid	539 (75.8)	25 (92.6)	14 (100)	20 (90.9)
Budesonide	30 (4.2)	4 (14.8)	3 (21.4)	3 (13.6)
Doxycycline	281 (39.5)	7 (25.9)	2 (14.3)	6 (27.3)
Azithromycin	288 (40.5)	8 (29.6)	2 (14.3)	5 (22.7)
Ivermectin	271 (38.1)	12 (44.4)	2 (14.3)	10 (45.5)
Tocilizumab	16 (2.3)	0 (0)	1 (7.1)	1 (4.5)
Itolizumab	2 (0.28)	0 (0)	0 (0)	1 (4.5)
Zinc	471 (66.2)	11 (40.7)	9 (64.3)	12 (54.5)
Other	73 (10.3)	2 (7.4)	3 (21.4)	2 (9.1)
Steroids				
Dexamethasone	290 (40.8)	8 (29.6)	6 (42.9)	6 (27.3)
Prednisone	37 (5.2)	3 (11.1)	1 (7.1)	4 (18.2)
Methylprednisolone	218 (30.7)	14 (51.9)	8 (57.1)	12 (54.5)
Antifungal treatment‡				
Amphotericin B	642 (90.3)	20 (74.1)	8 (57.1)	17 (77.3)
Posaconazole	426 (59.9)	13 (48.1)	9 (64.3)	13 (59.1)
Isavuconazole	27 (3.8)	4 (14.8)	1 (7.1)	5 (22.7)
Surgery	466 (65.5)	17 (63)	8 (57.1)	15 (68.2)
Voriconazole	9 (1.3)	1 (3.7)	5 (35.7)	2 (9)
Amphotericin treatment				
Amphotericin B deoxycholate	62 (8.7)	7 (25.9)	4 (28.6)	10 (45.5)
Liposomalamphotericin B	604 (85)	17 (63)	7 (50)	14 (63.6)
Amphotericin B lipid complex	113 (15.9)	1 (3.7)	4 (28.6)	5 (22.7)
Hospital course				
Hospitalized	423 (59.5)	21 (77.8)	14 (100)	17 (77.3)
Length of hospital admission, days	12.5	19.6	20.3	19.1
Intensive care unit admission	98 (13.8)	9 (33.3)	9 (64.3)	7 (31.8)
Level of oxygen requirement				
High-flow nasal cannula	82 (11.5)	4 (14.8)	4 (28.6)	3 (13.6)
Continuous positive airway pressure	6 (0.84)	1 (3.7)	0 (0)	0 (0)
Bilevel positive airway pressure	23 (3.2)	4 (14.8)	3 (21.4)	3 (13.6)
Ventilator	24 (3.4)	2 (7.4)	2 (14.3)	3 (13.6)
Extracorporeal membrane oxygenation	7 (0.98)	0 (0)	0 (0)	0 (0)
Hudson mask	103 (14.5)	2 (7.4)	1 (7.1)	2 (9.1)
Nasal prongs	133 (18.7)	4 (14.8)	1 (7.1)	5 (22.7)
Non-rebreather	65 (9.1)	7 (25.9)	6 (42.9)	5 (22.7)
No oxygen required	297 (41.8)	6 (22.2)	1 (7.1)	4 (18.2)
Outcome after treatment				
Incomplete recovery	345 (48.5)	11 (40.7)	4 (28.6)	4 (18.2)
Full recovery	264 (37.1)	11 (40.7)	8 (57.1)	13 (59.1)
Death	83 (11.7)	3 (11.1)	2 (14.3)	5 (22.7)
Lost to follow-up	18 (2.5)	1 (3.7)	0 (0)	0 (0)
Vision loss	243 (34.2)	7 (25.9)	3 (21.4)	7 (31.8)

* Values are expressed as n (%) unless otherwise indicated.

† Totals may add up to more than 100% because of coinfection by multiple fungi.

‡ Totals may add up to more than 100% because use of multiple treatment modalities.

All patients received both COVID-19 treatment and antifungal treatment (Table 2). For COVID-19 treatment, 556 patients (81.3%) received corticosteroids, 476 (69.6%) received zinc, 292 (42.7%) received azithromycin, 283 (41.4%) received doxycycline, and 274 (40.1%) received ivermectin. The distribution of antifungal therapy is detailed in Table 2.

Overall, the crude mortality rate was 11.3% for mucormycosis and 22.7% for mixed infections. No deaths occurred in the *Aspergillus* and candida groups. Full recovery was seen in 252 patients with mucormycosis (36.5%), 2 (18.2%) with *Aspergillus*, 1 (25%) with candida, and 13 (59.1%) with a mixed isolate. Vision loss was seen in a third of the patients but was not seen in the candida group.

## DISCUSSION

This much larger study validates the utility of an online registry to obtain high-quality data about a large number of cases in close to real time. Analysis showed confirmation of many known characteristics of COVID-19–associated mycoses: mostly mucormycosis (primarily rhinocerebral), with high morbidity and mortality^2^^,^^4^; increased representation of patients with diabetes and male sex^1^^,^^2^^,^^4^; a high incidence of hypertension (17.2%), as seen in studies of Covid-19–associated pulmonary aspergillosis^5^; and high corticosteroid use that likely increased the risk for fungal infections.^6^ This lends credence to other findings that deserve further study, such as the use of zinc, which possibly predisposed these patients to fungal infections.^7^

This large sample, which included cases from five countries, shows that COVID-19–related mycoses are not limited to India. Although mucormycosis has dominated recently published studies, this paper also describes fungal infections such as *Aspergillus* and candida underlying COVID-19.

Study limitations were previously described^2^: self-reporting resulting in unmeasured bias and missing cases. Furthermore, there was no control group without COVID-19 for comparison.

Further studies might investigate the burden of fungal infections in various nosocomial settings and equipment. Because much of this information was collected before vaccination was widely available in the countries studied, further studies could compare secondary mycotic infections in vaccinated and unvaccinated patients.

The goal of this study was to establish a global online registry that allows providers anywhere to report COVID-associated fungal infections in real time. The large sample size will allow further analysis of clinical characteristics associated with poor outcomes.

## Supplemental files


Supplemental materials
