# Supplementary figures and images for: Clinico-Epidemiologic Characteristics of Patients Reported in the Mycotic Infections in COVID-19 Registry
Source: Am J Trop Med Hyg. 2022 Dec 19;108(3):584–7. doi: 10.4269/ajtmh.22-0503 (PMC9978542; doi:10.4269/ajtmh.22-0503)

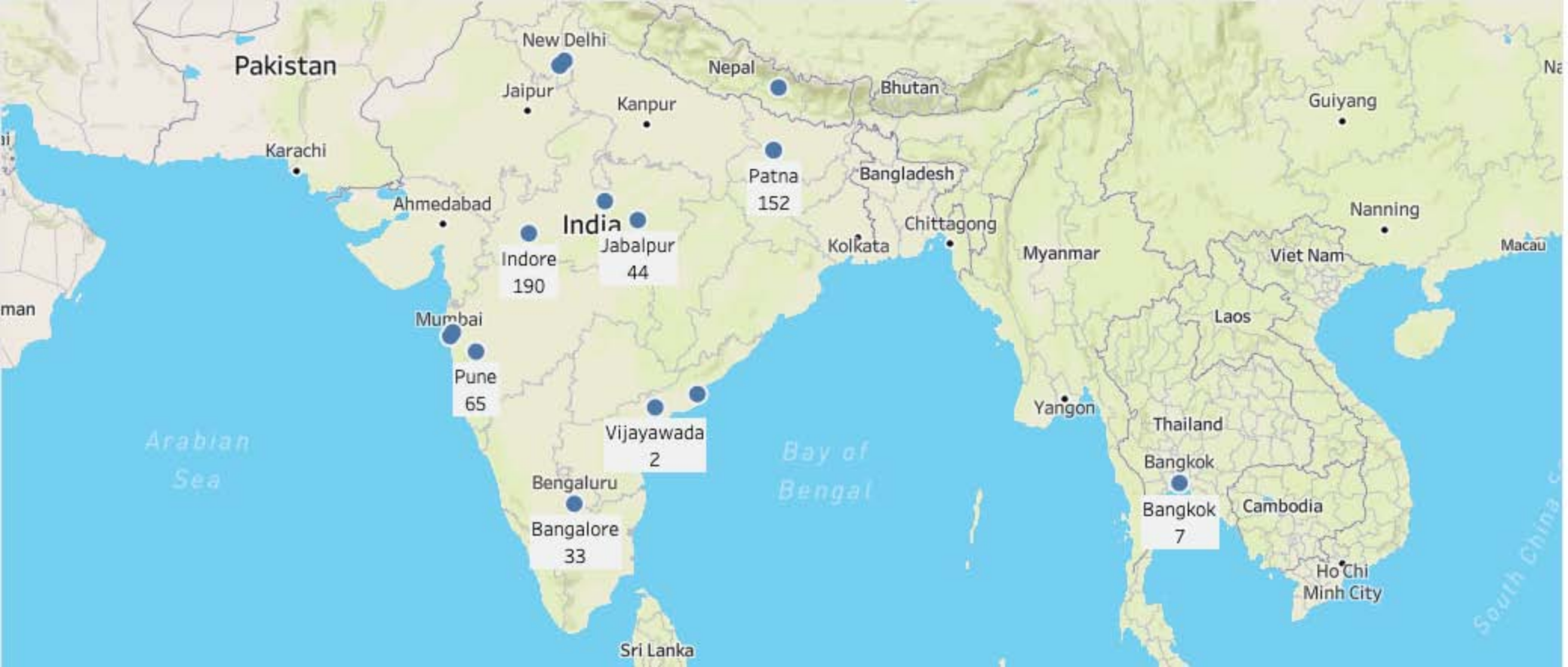

Supplement: Supplementary file 1 [file tpmd220503.SD1.pdf]
